# Neurobiological Processes Induced by Aerobic Exercise through the Endocannabinoidome

**DOI:** 10.3390/cells10040938

**Published:** 2021-04-17

**Authors:** Fabiola Forteza, Giada Giorgini, Frédéric Raymond

**Affiliations:** 1 Centre Nutrition, Santé et Société (NUTRISS), Institute of Nutrition and Functional Food (INAF), Université Laval, Québec, QC G1V 0A6, Canada; fabiola.forteza.1@ulaval.ca; 2 École de Nutrition, Faculté des Sciences de l’Agriculture et de l’Alimentation (FSAA), Université Laval, Québec, QC G1V 0A6, Canada; 3 Canada Excellence Research Chair in Microbiome-Endocannabinoidome Axis in Metabolic Health (CERC-MEND), Université Laval, Quebec, QC G1V 4G5, Canada; giada.giorgini.1@ulaval.ca; 4 Centre de Recherche de l’Institut Universitaire de Cardiologie et de Pneumologie de Québec (IUCPQ), Québec, QC G1V 4G5, Canada

**Keywords:** stress, physical activity, endocannabinoids, microbiome, depression, mental health, peripheral nervous system, central nervous system, brain

## Abstract

Evidence suggesting the triangulation of the endocannabinoid system, exercise, and neurological health is emerging. In addition to the endocannabinoids *N*-arachidonoylethanolamine (anandamide; AEA) and 2-arachidonoylglycerol (2-AG), the expanded endocannabinoid system, known as the endocannabinoidome (eCBome), appears to be an important player in this relationship. The eCBome includes several endocannabinoid-like mediators such as *N*-acylethanolamines and 2-monoacylglycerols, the enzymes involved in their biosynthesis and degradation, and the receptors they affect. This review aims to relate the functional interactions between aerobic exercise, and the molecular and cellular pathways related to endocannabinoids, in the hypothalamus, hippocampus, and the periphery, with special attention given to associations with emotional state, cognition, and mental health. Given the well-documented roles of many eCBome members in regulating stress and neurological processes, we posit that the eCBome is an important effector of exercise-induced central and peripheral adaptive mechanisms that benefit mental health. Gut microbiota imbalance, affecting the gut-brain axis and metabolism, also influences certain eCBome-modulated inflammation pathways. The integrity of the gut microbiota could thus be crucial in the onset of neuroinflammation and mental conditions. Further studies on how the modulation by exercise of the peripheral eCBome affects brain functions could reveal to be key elements in the prevention and treatment of neuropsychological disorders.

## 1. Introduction

**Figure 1:**
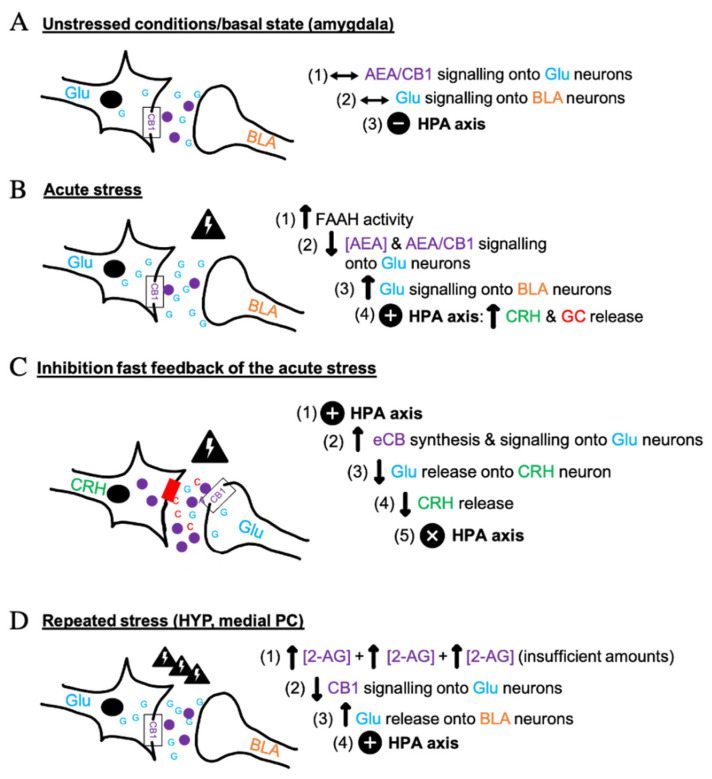
Interaction between endocannabinoids, glutamate, and the HPA axis under stressful conditions. This figure summarizes the theory presented by Hill et al. (2010) about the intervention of eCBs, the glutamatergic (Glu) system, glucocorticoids (GC) and the corticotropin-releasing hormone (CRH) in response to different stressful stimuli, the stress response being dependent on glutamatergic neurons and CB1 signaling in murine models. The synapses illustrated occur in the amygdala, hypothalamus (HYP), hippocampus or prefrontal cortex (PC). Symbols show the nature of the effect on the HPA axis, either neutral (−), excitatory (**+**) or inhibitory (**x**). (**A**) The non-stressed condition enables to maintain normal levels of AEA and to stabilize Glu activity onto the basolateral amygdala (BLA) neurons, which does not affect the HPA axis. Under acute stressful situations (**B**), the HPA is activated to ensure the survival of the organism. In response to stress, fatty acid amide hydrolase (FAAH) activation promotes the hydrolysis of AEA in all brain regions considered, whereas there are unchanged or lower 2-AG levels in the amygdala and in the HYP, respectively. The impaired AEA/CB1 signaling onto Glu synapses in turn induces a higher Glu input onto BLA neurons. A negative fast feedback occurs when there is a hypersecretion of cortisol (**C**). Biosynthesis of eCBs is then engaged to activate CB1 receptors in Glu terminals, thus diminishing or even suppressing Glu release and further CRH production in a retrograde manner. BLA-GABAergic outflow through eCB signaling can also generate a negative fast feedback in the amygdala. During chronic stress (**D**), the insufficient increases of 2-AG/CB1 signaling induced by many stressful circumstances downregulates CB1 signaling in the medial PC and the HYP that results in a “hypocannabinoid state”.

The interactions between stress and endocannabinoids (eCB) have been investigated in the past decade in parallel with the intensification of studies on the eCB system (ECS). A stress is generally defined as any stimulus that threatens the homeostasis of an organism, physiologically or subjectively [1,2]. Similar to a stress, physical exercise activates the ECS and stress-related endocrine pathways such as the HPA axis and catecholaminergic system, important for mood control and alertness [3,4] (Figure 1).

The coordination of the hypothalamus-pituitary-adrenal (HPA) axis, the sympathetic, and the parasympathetic branches of the autonomous nervous system is necessary in the face of a physiological or metabolic stress [5]. The sympathetic branch represents the “fight or flight” reaction and innervates the adrenal cortex, while the parasympathetic branch moderates this direct sympathetic response. HPA axis activation through eCB signaling in humans contribute to stress-induced affective disorders if the functions of the ECS are compromised [6,7,8]. Aerobic exercise, known to modulate neuropsychological behaviors, provides beneficial effects on brain functions [9] and could even counteract neuropsychiatric diseases such as schizophrenia through the intervention of the ECS [10]. The ECS is exactly implicated in neuronal processes such as analgesia, sedation, anxiolysis and sensations of wellbeing due to sustained aerobic physical activity [11,12].

The main objective of the present work is to provide an in-depth examination of ECS-mediated neurobiological mechanisms of aerobic exercise, with emphasis on its molecular and cellular aspects. In addition to these central functions of eCB signaling, many others have been described, including feeding, energy homeostasis, mood, learning memory, growth, development, and reward processes, which have mainly been investigated in rodent models. The peripheral ECS functional pathways discussed in this review are multiple and also include, among others, the regulation of inflammation and gut-barrier functions [13], considering gut microbiota also has a tangible impact on gut-brain communications as well as mental functions [14,15,16].

## 2. Overview of the Endocannabinoid System and Ensuing Endocannabinoidome

The ECS is a complex network where endogenous bioactive lipid signals are produced by cells and released “on demand”. They are later degraded by catabolic enzymes to modulate physiological functions in a space and time-specific manner. The core eCBs are anandamide (AEA) and 2-arachidonoylglycerol (2-AG), which belong to the *N*-acylethanolamine (NAEs) and 2-monoacylglycerol (2-MAGs) families, respectively [17]. These molecules were initially identified as endogenous ligands for the cannabinoid CB1 and CB2 receptors. CB1 is specific to appetite, nociception, adipogenesis and pro-inflammation whereas CB2 is anti-inflammatory [18,19] and reduces cytokine release [20]. The CB1 receptor is predominantly expressed in the mammalian central nervous system (CNS) [21,22] and at lower levels in non-neuronal peripheral tissues. The lower levels of CB1 receptors in peripheral cells are, however, not representative of its functional relevance in the organism [23]. The CB2 receptor is more prevalent in the periphery, particularly within immune cells [21] including those of the brain (microglia), but its expression in neurons and functions on anxiety behaviors are still enigmatic [24]. Because eCBs act on different receptors located in specific cell types, with vastly different biological activities, some of which produce and release different hormones and molecules, the eCBs are multifunctional and regulate, among others, energy balance, inflammation, and behavior [11,13,25,26]. They are pivotal molecules in synaptic excitatory (glutamatergic) and inhibitory (GABAergic) transmission of neurons and glial cell activity in the CNS [24,25] (Figure 1). CB1 and CB2 are not the only receptors whose activity are responsive to AEA and 2-AG, as both ligands are able to modulate TRPV1, PPARG, and, not without controversy, GPR55 [26].

While both 2-AG and AEA are derived from arachidonic acid containing phospholipids, they are synthesized and degraded by distinct enzymatic pathways, which impart fundamentally different physiological and pathophysiological roles to these two eCBs [27]. Indeed, the enzymes NAPE-PLD, ABDH4, GDE1, and PTPN22 contribute to the biosynthesis of AEA, and FAAH to its degradation. DAGLα and DAGLβ are involved in the biosynthesis of 2-AG while MAGL, ABDH6, ABDH12, and FAAH inactivate this molecule. Related bioactive NAEs and 2-MAGs, which vary from AEA and 2-AG by virtue of their fatty acids component, often share biosynthetic and metabolic pathways with eCBs, as is the case for palmitoylethanolamide (PEA), oleylethanolamide (OEA), and 2-oleylglycerol (2-OG) [7,28]. The role of eCBome mediators is dependent on their molecular targets, which are varied and include ion channels, G-protein coupled receptors, and nuclear receptors [26].

The eCBs and eCB-related lipids, with their receptors and their metabolic enzymes (which include those of the ECS), constitute an expanded ECS or endocannabinoidome (eCBome) [29]. Recent studies evoked how other NAEs, 2-MAGs, *N*-acyl aminoacids, *N*-acyl-dopamine/taurine/serotonin contribute to the system [30]. Some eCB congeners and non-eCB long chain fatty acid derivatives have been found to regulate functions that are controlled by eCBs, such as gut-barrier permeability [31], antinociception, appetite, the immune system, and cell biology in general [32], but do so via non-CB1 and non-CB2 receptors. Receptors that are considered to be part of the eCBome include PPARα, GPR18, GPR55, GPR119, Cav3, TRPV1, TRPV2, and TRPV4. Transient receptor potential vanilloid type-1 (TRPV1) channels, which are activated by both NAEs and MAGs, often plays opposite roles to those of CB1 [29]. Activation of the TRPV1 channel was found to improve exercise endurance [33]. Overall, the role of eCBome mediators, including AEA and 2-AG, depends on the receptor to which they attach to mediate cell-type and tissue-specific functions [17]. The function of several actors of the eCBome still remains nebulous, even more when considering the interaction between neurobiological processes and exercise.

## 3. The Molecular and Synaptic Processes of ECS in Stress

A growing body of studies established that the ECS has a major role in the modulation of the HPA axis [3] i.e., the core stress efferent axis [34,35], and of the catecholaminergic system during stress [4]. When a physiological response is induced under stressful conditions, the ECS has been suggested to mediate or participate in the regulation of its psychological/emotional consequences, impacting on motivation and mood [36]. Acute and repeated stress can cause pathologic anxiety levels if not coordinated properly through the ECS, glucocorticoids (GC) and Glu mechanisms in corticolimbic synapses, and sympathetic and parasympathetic nervous system synergy. This general function is exerted at the cellular and molecular levels through activation of CB1 receptors that inhibit the release of neurotransmitters, thus fine-tuning their actions. These receptors are activated by eCBs released from post-synaptic neurons, which finally allow a cellular response. As shown in Figure 1, the key neurotransmitters intervening in ECS-regulated stress synapses are glutamate (Glu) and gamma-aminobutyric acid (GABA). In the hypothalamus, sympathetic nervous system and adrenal gland products, catecholamines, and hormones such as corticotropin-releasing hormone (CRH) and GC, are mediated by eCBs [5]. Glu has a role in the activation of the acute stress response, as well as in learning and memory synapses. GABA acts as a primary inhibitor of such response in the CNS and as a reinforcer of the anti-anxiolytic effect of running in mice [37]. This effect occurs in the basolateral amygdala (BLA), a key anxiety/fear-mediating region [38]. CRH release from the hypothalamus followed by adrenocorticotropic hormone (ACTH) produced by the pituitary gland, one hormone stimulating the release of the other, also act as a main signal in the stress biological loop [6,34]. GC, such as cortisol in humans and corticosterone in rodents [9], are involved in the physiological response to stress. Their production occurs in the adrenal gland and correlates with AEA levels due to the ability of GC to quickly mobilize eCBs [2].

In non-stress conditions, a stable tone of AEA maintains stability in Glu signaling onto the BLA neurons, which provides a neutral effect on the HPA axis (Figure 1A). An acute stress results in the activation of the HPA axis and the release of GC [2,6,34] (Figure 1B). Initially, AEA concentrations are reduced because of the activation of the fatty acid amide hydrolase (FAAH), the main AEA catabolic enzyme [39]. This initial effect causes reduction of CB1 activity on glutamatergic neurons [3], thus allowing Glu release into the BLA nucleus. HPA activation, CRH and the final GC secretion result in a “fight or flight’’ stress response [6]. The contribution of 2-AG in stress behavior is less documented, but its levels also seem to decrease in the presence of acute stress [40], to be then either elevated or decreased throughout the corticolimbic stress circuit with repeated stress exposure [41]. The outflow of eCBs depends on the nature of the stressor (social, psycho-emotional, physiological), sex, genetics, fitness level, and affective status [42,43,44]. Subsequently to stress, BLA AEA-CB1 signaling becomes particularly important in the mitigation of stress-induced anxiety [38] and a behavioral habituation to stress can occur if CB1 signaling is maintained in an optimal range. In fact, the cortical CB1 attached to Glu neurons has dichotomic action, generating opposing anxiety-like phenotypes and showing its regulatory function in stress in mice [24]. As presented in Figure 1C, CB1 receptors act as “retrograde neuromodulators” in Glu and GABA terminals to eventually prevent CRH production [24]. Excitatory inputs stimulate Glu release and biosynthesis of eCBs in post-synaptic terminals, which activate pre-synaptic CB1, leading to the suppression of Glu release [24]. A similar mechanism occurs at GABAergic synapses. More precisely, the hyperstimulation of the HPA axis due to a novel situation may be reversed by an endogenous negative fast feedback through eCBs and BLA-GABAergic outflow, which inhibits neurotransmitter action [2,24,34,41]. Acute exogenous administration of corticosterone permits the restoration of eCB signaling and homeostasis following cessation of stress [34,43]. The recruitment of eCB signaling by GC is indeed important for BLA-GABAergic transmission, in the development of fear extinction, stress recovery and return to basal conditions, for which the molecular interactions are shown in Figure 1A [2,41]. Chronic stress may have distinct molecular effects as there may be a late phasic and limited 2-AG production in the medial PC and the HYP through hypersecretion of GC (Figure 1D) [41]. The subsequent downregulation of CB1 signaling results in a “hypocannabinoid state” that can be prevented by either the stress retrograde pathway (Figure 1C) or by CB1 agonists and indirect agonists that favor fear extinction and resilience to stress [24]. Chronic stress can be detrimental for human physical and psychological integrity. Current research suggests that eCBs have a dampening effect on the neuroendocrine circuits in the brain and the periphery in response to stress [3,34], suggesting that the ECS plays a role in stress coping by moderating stress-induced excitability [7].

## 4. Exercise and Endocannabinoids

Similar to stress, exercise is now accepted to act as a regulator of the ECS. The pioneering study of Sparling et al. (2003) reported an increase of AEA and 2-AG after 45 min of cycling or running [45]. Feuerecker and colleagues (2012) interpreted this finding in the way that the intensity of an acute physical activity would be responsible for AEA up-regulation [4]. Altitude would further enhance the effect of an intense exercise session on circulating AEA levels. The correlation between AEA and epinephrine after an exercise bout in high altitude corresponds to the well-documented catecholaminergic and ECS-modulated changes occurring during general stress [4,46]. By contrast, chronicity of exercise seems to be a factor underlying 2-AG reactivity to physical activity [4], but this statement is less powerful than for AEA. In fact, consistent with the concept that a repetitive stress affects human homeostasis (Figure 1D), 2-AG tone would also augment in the presence of repetitive physical stress and initiate stress-coping behaviors. However, regular aerobic exercise and unpredictable chronic stress (social, physical and psychological) do not affect AEA levels in the same manner, in the sense that the former raises AEA levels and the other increases FAAH and neuronal excitability, thus reducing AEA levels [9,38]. Additionally, prolonged psychological stress and sleep deprivation reduce BDNF and AMPK basal levels and inactivate BLA AEA/CB1 signals unlike aerobic exercise [9]. These maladaptive responses would affect spatial memory, and lead to stress-induced anxiety and neurological vulnerability, as in depression and schizophrenia, which would be rescued by regular exercise [38].

**Figure 2:**
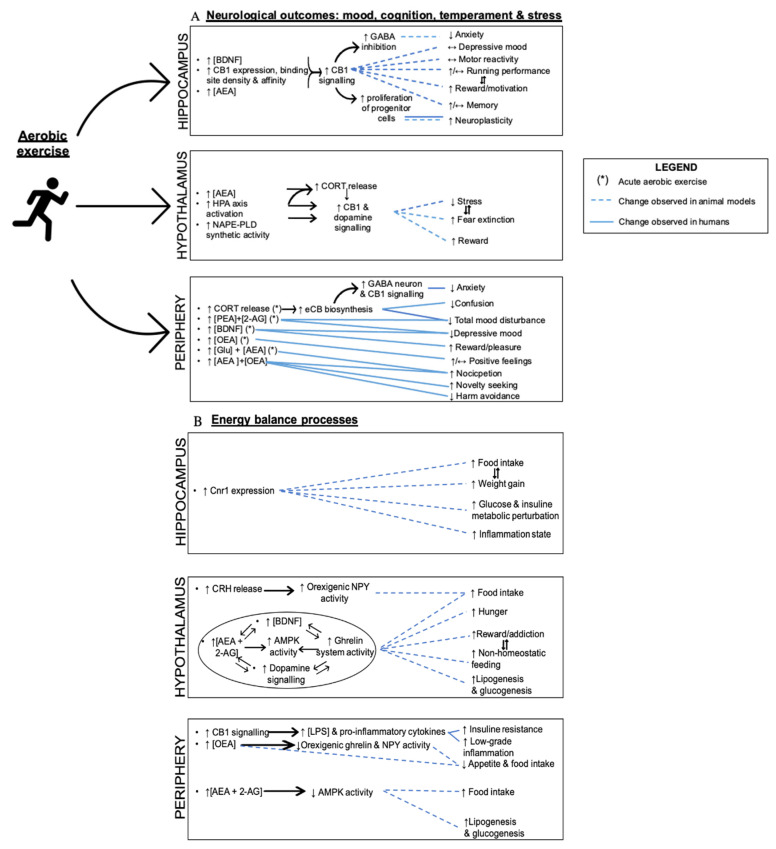
The possible ECS-related neurological outcomes of aerobic exercise and metabolic processes in hippocampal, hypothalamic, and peripheral tissues. The eCBs are involved in brain and exercise interactions. (**A**) Cross-sectional and prospective studies have been selected and used to summarize the implication of eCBs in mood, temper, cognition, and motor skills in relation to acute and chronic aerobic exercise. Looking at the hippocampus, aerobic exercise was associated with the increase of CB1 signaling and hippocampal BDNF expression (Ferreira-Vieira et al., 2014), exerting a neutral to positive impact into neuroplasticity and mental factors in animals. In the hypothalamus, the HPA axis and CB1 signaling reduce stress and fear. In the periphery, aerobic exercise seems to have positive impacts on the phenotype of human brain factors through the rise of OEA, AEA and a cortisol-AEA-GABA cascade. (**B**) The eCBs also impact on metabolism, thus playing a secondary neurological role. In the hippocampus, some animal studies suggested that CB1 overactivity would lead to inflammation and obesity-related consequences such as hyperphagia and glucose intolerance. The hypothalamic AMPK, eCBs, dopaminergic and ghrelin systems might interact to activate feeding, as well as CRH release. In the skeletal muscle, liver and adipose tissues of humans and animals, eCB/CB1 signaling would play the same role with possible inhibitory effects by OEA. CB1 signaling would also increase lipopolysaccharide (LPS) levels, low-grade inflammation, and insulin resistance. Physical activity and diet could be putative triggers of energy seeking molecular mechanisms and be implicated in inflammatory processes.

As exercise induces mental wellness, it promotes the expression of a broad variety of molecules, including BDNF [47]. BDNF is a member of the family of neurotrophins [48], which have been considered responsible for multiple neurobiological processes such as reward/addiction [49,50], oxidative stress, neuroinflammation, brain oxygen perfusion [9], GABAergic function, and exercise-induced neuronal plasticity, a well-established interaction in the hippocampus (Figure 2A). In vitro work has suggested an interaction between BDNF and ECS in the context of neurogenesis [51]. In a culture model of cerebellar granule neurons, BDNF was shown to regulate the expression of CB1 receptor and MAGL [52]. Moreover, in trained male cyclists, BDNF levels increased in a way similar to NAEs such as AEA, PEA, and OEA, and then slowly decreased during the recovery phase after 60 min of exercise at 55% of maximal power and intensive 30 min trial on an ergocycle [49]. The increase of AEA levels might have caused the rise of BDNF levels, whereas the increase in cortisol, also observed in this study, could have stimulated AEA biosynthesis [49]. Central and peripheral levels of BDNF, related to brain protection, is reactive to various physical exercises [53], although the dose-response needs clarification.

GABAergic system and ECS are tightly involved in the stress-mediated responses. Indeed, exercise and homeostatic stress would involve distinctive, but concomitant mechanisms of eCBs, BDNF, and GABA. GABAergic synapses, CB1 and eCB metabolism are potent and valuable targets to treat stress-associated abnormalities, as suggested by a study from Rossi et al. (2008): GABAergic inhibition by CB1 was reduced after a single stressful episode and totally eliminated after repeated psychoemotional stress in the area of motor, cognitive, and emotional functions i.e., the striatum [54,55]. Wheel-running would serve to increase the inhibitory GABA receptor mechanisms in the ventral hippocampus in response to high aggression and tone down the excitatory circuits that might otherwise produce an anxiogenic state [37]. Under low stress conditions, the GABAergic flow may indirectly activate a greater number of neurons and connections and improve cognition [37].

The newer members of the eCBome may also play a role in the biology of exercise, but their functions require more investigation. Relevant findings include the potential role of GPR55 in the regulation of physical activity. Gpr55^−/−^ mice showed decreased physical activity compared to wild-type, leading to increased susceptibility to obesity [56]. GPR55 activation reduced anxiety in a murine model, suggesting that it may play a role in the anxiolytic activity of exercise, given exercise increases its eCBome lipid ligands [57]. Indeed, exercise interventions were shown to modulate PEA, a ligand of GPR55, in the muscle of women with chronic neck shoulder pain [58]. In a randomized controlled trial, oral doses of PEA improved several markers associated with muscle recovery after exercise [59]. These studies support the idea that exercise increases a GPR55 agonist, which could potentially link back exercise anxiolytic activity to eCBome-mediated activation of GPR55.

In addition to the functions of eCBome proteins, their transcriptional regulation is also important, albeit complex. Indeed, the eCBome may respond dependently to different types of exercise, as shown in a study of gene expression in the skeletal muscle where several eCBome enzymes and receptors were modulated differentially by resistance and aerobic exercise [60]. For example, receptors GPR55 and GPR119 were upregulated after aerobic exercise, but not after resistance exercise. By contrast, FAAH1 gene expression was downregulated in aerobic and resistance exercise, both acute and chronic. In a rat model, long-term exercise increased the liver expression of PPARα, which correlated with the improvement of metabolic parameters [61]. Given that transcriptional differences between tissues can be associated to diverging gene interaction networks [62,63], the complexity of eCBome response to exercise or stress is staggering.

## 5. Brain Endocannabinoidome Signaling

Endocannabinoids AEA and 2-AG are biosynthesized in CNS areas such as the prefrontal cortex [42], the hypothalamus, the hippocampus, and the amygdala, among others. A direct feedback-regulated circuit [24] through GC-mediated pathways during stress [1] mediates eCB signaling in these central structures. eCB signaling and endocrine HPA axis activity are a response to all stressful stimuli, as shown by a series of in vitro and in vivo studies [2]. Multiple evidence indicates that central and peripheral triggers activate eCB/CB1 signaling to regulate neurotransmitter release in order to bring it back to that of non-stressful conditions and inhibit stress response through GC mechanisms [1,41] (Figure 1A).

### 5.1. Hippocampal Endocannabinoidome

The hippocampus is recognized as a key brain region for cognitive and mnemonic processes, and has a central role in neurogenesis [8,64], thus contributing to neuroplasticity and energy balance. eCBs intervene in mood, depression, and stress hippocampal functions [65]. Hippocampal eCBs signaling is sensitive to stimuli such as exercise [8,65]. The neurological processes and outcomes of exercise on hippocampus are represented in Figure 2A.

Hippocampal CB1 was shown to be a key element in wheel-running distance and coordinated locomotion but was not sensitive enough to chronic running to induce changes in reactivity and depression-like behaviors in wild type mice compared with knockout ones [11,64]. Indeed, during a six-week wheel-running protocol, CB1 receptors were found to control running distance in mutant mice (decrement of 30–40%) but neurogenesis increased to a similar extent (37–39%) in both CB1 knockout (CB1^−/−^) and wild type CB1^+/+^ models [64]. Nonetheless, the wheel-running protocol could have a positive influence on fear in CB1 knockout mice by counteracting the deficiency in fear extinction in these mice through fear memory reduction [64]. In sum, the contribution of eCBs/CB1 in motivation in the hippocampus can be translated into higher total free-running distance in rodents [66], possibly through circulating corticosterone levels and reward-driven behaviors [64]. A lesser volume of voluntary exercise (eight-day intervention) increased CB1 binding density and affinity, and augmented AEA levels in the hippocampus [8], without any effect on the total running distance. Administration of CB1 antagonist AM251 revealed that exercise-induced nervous cell proliferation in the dentate gyrus of the hippocampus would be dependent on the elevation of AEA/CB1 signaling [8]. In the experiment of Ferreira-Vieira and coll. (2014), one week of treadmill running was enough for the ECS to enhance spatial memory in mice, which was potentiated with the treatment with FAAH inhibitor URB597 [67]. Gamelin et al. (2016) showed that a high fat diet (HFD), exercise, and HFD combined with exercise provoked a significant increase in CB1 receptor gene expression in the hippocampus compared to controls [65]. Hippocampal CB1 overactivity was suggested to participate in food intake, weight gain, and glucose metabolism perturbation following exercise or HFD feeding in rats (Figure 2B). In the context of physical exercise, it is important to mention that upregulation of brain eCB/CB1 signaling induced by exercise does not necessarily occur in those circuits where it would activate food intake and inflammation despite the association of CB1 with obesity.

To sum up, physical activity and its impact on eCB signaling in animals produce a yet to be fully understood impact on running performance, motivation, memory, and neuroplasticity, through hippocampal CB1 signaling. CB1 could also play a role in those metabolic-driven inflammatory processes and excessive weight gain that extensively impact on neural functions [68].

### 5.2. Hypothalamic Endocannabinoidome

The hypothalamus is a major site of interaction between eCBs, hormones, neuropeptides, and neurotransmitters for many pathways in energy balance [69]. Previous studies demonstrated that phytocannabinoids, consumed for medical or recreational purposes, altered dopaminergic activity in the medial forebrain bundle, passing through the hypothalamus, with effects on the brain reward system and addiction [11]. The hypothalamus, by being a core structure of the HPA axis, participates in reward and addiction processes as well as stress hormone pathways [4,35]. In fact, HPA axis hyperfunction is directly related to stress [14]. Acute or chronic exercise are forms of external stress that may contribute to HPA axis stimulation, thereby affecting mood and mental processes [17].

Inhibitors of the *N*-acylphosphatidylethanolamine phospholipase D (NAPE-PLD) enzyme, the principal NAE biosynthetic enzyme, can enhance HPA axis activation and impair fear extinction [70]. Hill et al. (2010) attested that fear extinction can be seen as a similar phenomenon as habituation to stress, which strengthens the idea that such a process is mediated by the ECS [2,71] and may be influenced by physical exercise (Figure 2A). On the other hand, the role of the hypothalamus in appetite and satiety is well known [17]. Ghrelin acts on this brain area to stimulates the appetite and food intake, and elevates fat deposition [72]. In fact, the implication of adenosine monophosphate activated protein kinase (AMPK) activity in feeding and metabolic functions within the hypothalamus has been extensively studied with regards to the tight collaboration between the ghrelin and ECS [72]. Acting as a resource sensor, AMPK is known as an intracellular enzyme regulating appetite and energy processes when ATP is in deficit [73]. The ghrelin system works independently and collaboratively with the ECS in the hypothalamus as well as in the ventral tegmental area in the midbrain to modulate energy balance and “non-homeostatic” appetite i.e., motivational processes to seek food rewards. The ghrelin system, neuropeptide Y (NPY) and the ECS activate the hypothalamic AMPK but inhibit adipose and liver AMPK activity to promote weight gain, adiposity, and food intake, and more precisely, lipogenesis and gluconeogenesis [72] (Figure 2B). Elevated AEA concentrations in the hypothalamus increase feeding via CB1 stimulation by modulating orexigenic and anorexigenic signals and by facilitating dopamine signaling [65], while OEA was found to reduce ghrelin and NPY activity in the periphery and to inhibit the orexigenic action of arachidonic acid-derived eCB AEA and 2-AG in adipose tissue [74].

Concentrations of 2-AG are 100 to 200 times higher than those of AEA in the brain [11,75] and this eCB is considered as a full agonist of both CB1 and CB2 receptors [4]. After HFD, elevation of 2-AG levels in the rat hypothalamus, but not in hippocampus, was mitigated by 12-week forced treadmill activity at moderate intensity (70–80% of maximal aerobic velocity) [65]. The authors concluded that hypothalamic 2-AG signaling is strongly involved in sport-induced energy expenditure and thermogenesis, which could lead to mood and stress management changes. Thus, the ECS can generate neuronal alterations in response to nutrition or physical effort also in the hypothalamus and the HPA axis. An imbalanced diet can also be a trigger to instigate inflammatory and obesity-like molecular patterns in nervous cells [5,15,76]. The hypothalamic eCB signaling induced by exercise may not only govern synapses leading to stress management and pleasure production, but ones of feeding behavior and reward considering its role in replenishing energy through food intake.

## 6. Peripheral Endocannabinoidome

### 6.1. Mood, Depression, and Anxiety

eCBs have a definitive role in cognition, motivation, and emotional states such as fear and stress anxiety, which are affected by physical exercise and other activities. In young adults, Brellenthin et al. (2017) detected a positive correlation between peripheral AEA concentrations post-exercise and the improvement of anxiety, total mood disturbance and confusion with preferred (self-determined) or prescribed acute aerobic demand [77]. The beneficial changes in mood that correlated with eCBs were greater in the preferred condition. There was a weaker increase of 2-AG and PEA levels following the exercise that was associated with the reduction of depression, tension, and total mood disturbance [77]. Concurring to the data of Brellenthin et al. (2017) in healthy adults, an experimental study among a group of women with major depressive syndrome showed negative correlations between AEA and 2-AG levels measured following prescribed aerobic exercise at moderate intensity and depressive mood [76]. In a study carried out with nine postmenopausal female choristers in the UK, scientists determined that activities other than running (singing, reading, cycling, and dancing) were related to improved emotional responses and hunger [78]. Levels of AEA and feelings correlated with each other as well as OEA and positive feelings. Cycling (non-mentioned intensity) immediately increased OEA levels (26%), decreased appetite, and had no effect on mood. Singing remained the activity with the most effects increasing AEA by 42%, PEA by 53%, and OEA by 34% with positive effect on mood and emotions. Considering the emotions felt as part of the physical, intellectual, or artistic activity experience, AEA and OEA have been shown to be consistently related to positive mood (Figure 2A, periphery). The difference in mood with specific amount, frequency, and intensity of exercise is still an area of research that needs further examination.

### 6.2. Motivation

The ECS might exert a control on neurological rewards, motivation and willingness that could promote intensive physical activity and physical performance [12,64]. In a bank vole rodent model, alteration of eCB signaling did not cause an increase in high-intensity voluntary running and swimming compared to forced physical activity, as it was hypothesized [79]. However, AM404, an eCB cellular reuptake inhibitor, decreased VO_2_swim, which suggests the involvement of eCBs in pathways controlling temporary motivation to locomotion i.e., the instantaneous willingness to undertake physical activity, and thus, aerobic performance [79]. The phasic effects on motivation observed in animals could be prolonged and even identified as forged temperamental traits within a longer time frame in humans. In a crossover study of Fernandez-Aranda et al. (2014), metabolism, eCBs, and physical activity have been linked with long-term psychological profiles in 189 obese and non-obese women [80]. Regular moderate-vigorous physical activity (lower in obese groups) had a linear association with circulating AEA and OEA levels. Temperament traits such as novelty seeking, and harm avoidance had respectively positive and negative relationships with exercise. There are some assumptions about CB1 involvement in exercise-related motivation in the periphery as in the CNS. Peripheral levels of AEA and OEA measured following frequent physical activity might be potentially related to certain healthy mental traits as represented in Figure 2A [80]. The molecular and mechanistic explanations beyond the correlations remain vague.

### 6.3. Inflammation

Energy imbalance can lead to chronic inflammation and metabolic disturbances such as hypertension, type 2 diabetes, and obesity [81]. These problems are tightly connected with ECS activity in systemic and central networks, and with the neuropsychological state (Figure 2B). The results of Grunewald et al. (2019) with rats point out CB1 as a contributor of lipopolysaccharide (LPS)-mediated low-grade inflammation [82]. The aggravation of inflammation and insulin resistance in the presence of LPS and CB1 agonists, and the attenuation of LPS inflammatory effects by CB1 inhibition allowed to conclude that blocking this receptor could lead to beneficial manipulation of metabolic endotoxemia [82] and to gut-barrier permeability [23]. In addition, overactivity of the CB1 signaling through high eCB levels contributes to insulin resistance, reduced substrate oxidation in the muscle, visceral adipose tissue and liver, and increased fatty acids and triglyceride accumulation, leading to obesogenic and inflammatory status in the long term [49]. Obesity is hence associated with a hyperactive ECS and aerobic exercise also increase plasma AEA levels. The administration of a sufficient dose of AEA, partial agonist of CB1 receptors, may however activate positive metabolic effects such as glucose uptake and mitochondrial biogenesis because of its faculty to bind to other receptors than CB1 [49]. Indeed, the bimodal effect of eCB signaling in feeding behavior can either promote energy storage and fat accumulation for survival, facilitating the consumption of rewarding and palatable food, or conduct to a lack of appetite depending on the tissue concerned and the dose of eCBs [23]. Even if CB1 activation in the hypothalamus has been previously associated with orexigenic effects, specifically with the inhibition of energy expenditure in obese and older animals, this statement is not as accurate when assessing CB1 signaling counter-effect on neuroinflammation in lean animals [25]. Chronic physical activity could help control CB1 expression favorably by promoting HPA maintenance and immune homeostasis [25,60].

### 6.4. Nociception

Antinociception require upregulation and stimulation of both CB1 and CB2 receptor, centrally and systemically. Two studies by Galdino and coworkers (2014) showed that both aerobic exercise and acute resistance exercise induced antinociception in rats in a rapid manner, lasting up to an hour after the exercise in another study [83,84,85]. Similarly to opioids, eCB signaling with exercise would reduce perception of pain and favor calmness through proprioceptive stimuli in the muscle and a parallel change in Glu neurotransmission in the brain [83,84]. eCB-mediated chronic pain would decrease with exercise, and potentially more with chronic exercise, through temporal changes in chronic inflammation and analgesia [83,84,85]. In a group of mice performing a single bout of treadmill running, exercise reduced nociceptive responses without impacting on inflammatory nociception, increased AEA and CB1 contents, and reduced FAAH expression [73]. CB1-antagonist AM251 reversed exercise-induced antinociception but, when combined with AMPK activation, restored the positive effect of exercise by pain inhibition. Thus, AMPK is not only implicated in energy metabolism but also acts as an intermediate between eCB/CB1 and exercise-induced analgesia [73].

**Figure 3:**
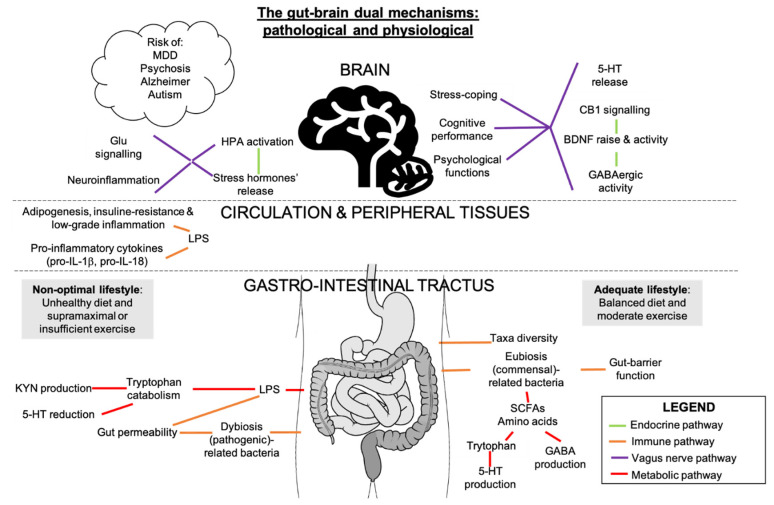
Gut-brain axis routes in mental health. The gut microbiota and the CNS are connected through immune, vagus nerve, metabolic and neuroendocrine-mediated pathways. These processes can all contribute to mental health or illness where eCBs and other molecules interfere depending on the cell or tissue. SCFAs are primary products of the fermentation of complex fibers by gut microbes in an intestinal balanced environment i.e., eubiosis. SCFAs induce GABA and serotonin (5-HT) production, attenuating depressive-like behavior. These neurotransmitters can also exert their roles in the brain where, with BDNF and CB1, they can bring positive mood changes. On the other hand, LPS-mediated low-grade inflammation in the gut caused by disruption of microbiota composition (dysbiosis) and intestinal barrier perturbation, would induce tryptophan degradation, raising kynurenine (KYN) levels and reducing 5-HT release, two events associated with depressive mood. The action of pro-inflammatory interleukins (pro-IL) and LPS would evolve in an inflammatory state in the brain. Inflammatory stimuli and stressor exposure would engage HPA overstimulation. HPA function through GC and ACTH circuitry release in gut microbiota-mediated anxiety-like behaviors is notable (Kang et al., 2014). Strong bidirectional correlations are found between major depressive disorder (MDD), psychosis, Alzheimer’s disease, autism spectrum disorder and intestinal dysbiosis, which correlates with the dietary and exercise patterns.

Despite these reports, a recent review has highlighted many gaps in our knowledge of how the ECS participates in controlling pain under an exercise-rich lifestyle [21]. In the study of Stensson and Grimby-Ekman (2019), a 30 min arm-cycling intervention significantly altered the relationship between plasmatic AEA and Glu levels in the chronic pain group compared to the pain-free group [86]. This conclusion indicated that the ECS and glutamatergic pathways are not just altered with stressing stimuli but also with physical exercise, where they play a major role in the perception and transmission of pain [86], as indicated in Figure 2A (periphery section). In addition, the possible mechanisms of headache prevention by exercise would include the elevation of eCB and serum brain-derived neurotrophic factor (BDNF) levels, which would reduce neuroinflammation and improve brain oxygenation [9], and beta-endorphin, which is presumably implicated in reward and pleasure post-exercise. The etiology of migraine could be linked to an “eCB deficiency syndrome” [43], as with fibromyalgia and other psychological pain disorders, which is consistent with stress-induced eCB deficits. Some lifestyle modifications, such as routine physical exercise, could reasonably upregulate ECS activity in the same way acetaminophen seems to elicit eCB signaling in rodents [43], and subsequently improve analgesia. Ultimately, energy homeostasis and systemic inflammation are strongly implicated in neurophysiology and neurodegeneration, including CNS disorders (Figure 3), through an interconnection of the central and the peripheral ECS with various molecules (LPS, AMPK, Glu, BDNF) (Figure 2). The inflammatory, metabolic, and pain status can reciprocally nurture mental and neurodegenerative disorders [87], as they have common molecular mechanisms [25]. Some of the mentioned diseases have been associated with changes in gut microbiota composition [88].

### 6.5. Stress-Associated Comorbidities

Exercise can modulate the ECS as a nonpharmacological treatment of comorbid stress-induced and mental issues [43]. Raichlen et al. (2012) documented a positive correlation between exercise-induced eCB signaling and beneficial psychological adaptations [12]. Depressive and post-traumatic stress disorder (PTSD), a widespread neuropsychiatric disorder resulting from a traumatic and stressful event, is a relevant example of a downregulated eCB function accompanied by abnormal fear, memory, cognition and mood processing [89]. The baseline peripheral concentrations of eCBs and eCB-like mediators (i.e., AEA, PEA, OEA, and 2-AG) are distinctly lower than in healthy controls without PTSD or depression [6,90], and thus, the reaction to stress may be different [44]. Chronic pain is another collateral effect of PTSD that could be blunted by eCBs such as AEA and 2-AG, which mediate analgesia in several animal models [91]. Crombie and colleagues (2018) confirmed that PTSD adults, females in majority, experienced significantly greater mood improvements and pain reduction following a moderate-intensity aerobic exercise lasting 30 min in relation with an increase of circulating 2-AG levels in comparison to the control group [20]. A reduction of negative mood with higher blood concentrations of 2-AG was likewise reported in Brellenthin et al. (2017) study in healthy individuals, whereas an increase in AEA was associated with vigor, indicating that a defective ECS in PTSD patients and a functional ECS in healthy people are activated and have favorable consequences on psychological behaviors following exercise [77]. However, these outcomes can be elusive if there is no consideration of the duration of mood improvement after the cessation of exercise. Exposure to social stress results in a significant increase of blood 2-AG levels as in the case of a sample of women, where both PEA and OEA declined aggressively under baseline during the stress recovery phase, for which the magnitude of these effects were equal for both depressive participants and healthy matched controls [7]. Based on these data, exercise seems to produce eCB-associated mental protection and antinociception relatively to stress. Physical activity and other stressors do not share all the same neurological pathways, but similar cells and molecules are involved: GABA, BDNF in corticolimbic structures, as mentioned beforehand, AEA/CB1, and 2-AG/CB1. These mediators would help to manage stress and be partly responsible for the natural anti-anxiolytic effect of exercise.

## 7. Potential Interactions between Gut Microbiota, Inflammation, and Endocannabinoidome in Mental Conditions

The gut microbiota is an ecosystem of bacteria that live along the enteric tract. It has multiple functions in homeostasis and, like the ECS, adapts in function of stressful stimuli. The bidirectional gut microbiota-brain communication has been suggested to influence stress, pain, impulsivity/decision-making alterations [92], mood disorders [93] and cognitive vulnerability [94,95] via immune, neuroendocrine/transmitters, neural and metabolic pathways [88] (Figure 3). These outcomes could be due to interactions between eCBs and gut microbiota, for example, through changes in the nutrient and caloric intake, which are modifiers of the gut microbiota and the ECS [43,96] (Figure 3). Absence of gut microbiome in germ-free mouse model was associated to neurophysiological deficits and altered levels of eCBs in the brain, which was partially rescued by fecal microbiota transfer (FMT) [97]. FMT of conventionally raised mice to germ-free mice induced changes in intestinal eCB levels and eCBome gene expression, strengthening the link between gut microbiota and the eCBome [98]. Moreover, FMT from mice with unpredictable mild chronic stress to naïve mice showed a transfer of depressive phenotypes that was concomitant with a decrease in eCBs and peripheral levels of fatty acids [68]. Studies relating to the relationship between diet, gut microbiota, and plasma eCBs showed correlation between several gut microbes and circulating eCBs, which was also influenced by fat mass and fatty acid intake [99,100].

Bacterial LPS could be involved in the relationship between the gut microbiome and the body, as LPS are inflammation markers and gate openers associated to intestinal permeability. Indeed, an LPS-eCB/CB1 regulatory loop influences the gut microbiota activity and barrier function through CB1 role in adipocyte and enterocyte physiology [23]. Increased consumption of meat proteins and saturated fat, characteristic of western diets, would diminish health-associated taxa abundances, induce an increase in serum LPS, associated with a rise of CB1 receptors, and eventually generate adipogenesis [96]. On the other hand, Mediterranean diet-related microbiota would correlate with reduced frailty in older adults, reduced inflammation, and improved cognitive function [101]. Dietary fibers, including prebiotics, which are fermented by bacteria and metabolized into short-chain fatty acids, block CB1 signals in adipose tissue and reduce fat mass, which provide further evidence of a microbiota-regulated eCB activity in metabolic syndrome [23]. These complex plant-based polysaccharides can control neuromodulators such as serotonin (5-HT), dopamine, GABA, cortisol, noradrenaline, tryptophan (the precursor of 5-HT), and HPA secretory products in athletes [5]. Dopamine, GABA, serotonin and BDNF can all be influenced and manipulated by gut microbes and are perturbed in germ-free rodents, which exemplifies the potential role of the gut microbiota in neurological functions [53]. It has also been suggested that “psychobiotic” strategies, such as FMT, administration of selective probiotics and prebiotics selected based on their anti-depressant potential [88,102] and their modulation of CB1 [43], could decrease neuropsychiatric symptoms and lead to pioneering personalized-preventive psychiatry [103].

Intensified and prolonged exercise stimuli were suggested to be deleterious for intestinal permeability, creating shifts in the metabolic profiling and functions of the gut microbiota [104,105], accelerating the appearance of neuropathologies [106]. In fact, strenuous exercise, with manifestations similar to overtraining, increases stress hormones and LPS translocation outside the gut to surrounding tissues, thereby increasing pro-inflammatory cytokines and intestinal permeability, inflammation, and gut permeability being inseparable [107], and declining GABA-mediated inhibitory homeostasis [5]. A physically demanding military environment would change gut microbiota composition and decrease gut-barrier functions associated with the enhancement of inflammation markers among young adults, independently from the diet [108]. Additionally, 23% of stool metabolites following prolonged physiological stress were linked to changes in gut microbiota composition [108]. An excessive physical demand can thus oppose the intestinal and mental adaptations of an adequate amount of exercise and can lead to cognitive susceptibility and physical performance decrement [108]. In opposition to excessive and overintense exercise, regular physical activity has been shown to affect the gut microbiota by inducing taxa that can beneficially affect metabolism, inflammation, cognitive outcomes [109], and chronic pain [84,85].

The review of Madison and Kiecolt-Glaser (2019) treats human-bacteria studies and more precisely, the health-prejudicial and health-promoting bottom-up pathways from gut to brain, a field of research that accelerated in the past two years but needs more longitudinal studies of larger sample size [15,93]. Obesity-related systemic inflammation correlated with changes in the gut microbiota ecosystem, short-chain fatty acids (SCFAs) production, which are key players in the gut-brain axis and adjuvants in the anti-depressant function, and alterations in metabolic pathways of tryptophan and kynurenine (KYN), a tryptophan derivative associated with depression [110] (Figure 3). Hence, gut bacteria would induce mood changes and brain functions via certain neurotransmitters and could indirectly influence eating behaviors, through the vagus nerve and the intestinal immune system [14,15,91] (Figure 3). In general, a positive balance of commensal/pathogenic bacteria proportions and diversity of gut communities are critical for gut health and aspects of brain function [109]. Non-strenuous exercise and a diet full of omega-3, micronutrients, prebiotics, quality of amino acids (precursors of neurotransmitters), and fermented food would confer positive cerebral outcomes and prevent the appearance of neuropathologies [109]. The gut microbiota could be an intermediary between eCB-mediated energy balance, inflammation, and brain-related functions [68].

## 8. Conclusions

There is a plethora of molecular pathways that control mood, cognition, and mental wellbeing. Central and peripheral ECS-induced processes and outcomes of aerobic exercise on cognition—including neurogenesis and neuroplasticity—motivation, mood/temperament, nociception, neuroinflammation, and mental health involved similar molecules as homeostatic stress pathways occurring in the CNS.

The brain and neurons are respectively the most important organ and cells involved in eCB-modulation of neurobiological factors and stress, the hypothalamus and hippocampus being particularly key areas. The main arachidonic acid-derived eCBs, AEA, and 2-AG, have specific functions according to the nature of the receptor and the cell-type of the receptor they connect to. The signaling of the receptor, CB1 for neurons, can have dual actions on feeding and anxiety-like behaviors and are thus responsible for the modulation of metabolism, stress or fear reaction/habituation, and mental functions. Cognition is slightly increased through the neuroplasticity induced by aerobic exercise, but the contribution of the ECS in memory improvement is still controversial. Motivation to exercise is enhanced through reward/addiction system stimulation, in which eCBs participate. The eCBs and aerobic exercise improve the variables of mood, temperament and antinociception and lessen anxiety/tension and depressive feelings caused by stress. When diet and exercise are not adequate, the metabolic mechanisms regulated by eCBs would most likely enhance lipogenesis and low-grade inflammation, and progressively, neuroinflammation and mental function alterations, with the gut microbiota exerting a crucial role. Chronic and mild aerobic exercise seems to generate neuroprotective, even neurorestorative effects, and to counteract the metabolic impairments that eCBs may induce in certain conditions. Still, the role of the extended eCBome in response to stress and exercise remains to be thoroughly investigated.

During aerobic exercise, the effects of eCBs on physical performance and locomotion appear to be minor, either neutral or slightly positive, at least judging from the articles selected for this review. Further detailing of the parameters of exercise (intensity, load, frequency, type, duration) that can mediate the neuroprotective efficiency could be helpful to develop strategies to improve athletic performance or optimize non-pharmaceutical health procedures. Considering that nutrition was not the main focus of this review, but still has a crucial value in eCBome modulation and mental health, future studies could be dedicated to the effect of specific dietary habits shifts and restrictions on neurological health, pain, and inflammation management. Moreover, we know that the eCBome influences metabolism and many other physiological systems, but it would be instructive to refine our understanding on how physiological systems can modify the eCBome in their own terms. To investigate which physiological mechanisms (oxidative stress, skeletal muscle activity, mitochondrial biogenesis, etc.) cause fluctuations in eCBome and other associated neuromodulators with exercise, could be another way to dig in the biological significance of the ECS-exercise interaction. Understanding how the modulation of peripheral eCBome by lifestyle affects the eCB functions in the hypothalamus and hippocampus would provide crucial insight on neuropsychological disorders.

## Data Availability

Not applicable.
